# The Influence of Hydrolytic Enzymes on Tannin Adsorption-Desorption onto Grape Cell Walls in a Wine-Like Matrix

**DOI:** 10.3390/molecules26030770

**Published:** 2021-02-02

**Authors:** Andrea Osete-Alcaraz, Encarna Gómez-Plaza, Pilar Martínez-Pérez, Florent Weiller, Julia Schückel, William G.T. Willats, John P. Moore, José M. Ros-García, Ana B. Bautista-Ortín

**Affiliations:** 1 Department of Food Science and Technology, Faculty of Veterinary Science, University of Murcia, Campus de Espinardo, 30100 Murcia, Spain; andrea.osete@um.es (A.O.-A.); mpilar.martinez2@um.es (P.M.-P.); jmros@um.es (J.M.R.-G.); anabel@um.es (A.B.B.-O.); 2 Department of Viticulture and Oenology, Faculty of AgriSciences, South African Grape and Wine Research Institute, Stellenbosch University, Matieland 7602, South Africa; florent@sun.ac.za (F.W.); moorejp@sun.ac.za (J.P.M.); 3 Department of Plant and Environmental Sciences, Faculty of Science, University of Copenhagen, DK-1001 Copenhagen, Denmark; js@glycospot.dk; 4 Glycospot R&D, Thorvaldsensvej 40, B102, DK-1871 Frederiksberg, Denmark; 5 School of Agriculture, Food and Rural Development, Newcastle University, Newcastle-upon-Tyne NE1 4LB, UK; william.willats@newcastle.ac.uk

**Keywords:** grape, cell-wall, tannin, polysaccharide, hydrolytic enzymes

## Abstract

This study evaluates the capacity of four hydrolytic enzymes to limit the interactions between grape cell-walls and tannins and/or to favor tannin desorption. Adsorption and desorption tests were conducted by mixing a commercial seed tannin with purified skin cell-walls from Syrah grapes, in the presence or absence of hydrolytic enzymes, in a model-wine solution. The effects of the enzymes were evaluated by measuring the tannins in solution by High Performance Liquid Chromatography (HPLC) and the changes in the cell wall polysaccharide network by Comprehensive Microarray Polymer Profiling (COMPP) while the polysaccharides liberated from cell walls were analyzed by Size Exclusion Chromatography (SEC). The results showed that the enzymes limited the interaction between tannins and cell walls, especially cellulase, pectinase and xylanase, an effect associated with the cell wall structural modifications caused by the enzymes, which reduced their capacity to bind tannins. With regards to the tannin desorption process, enzymes did not play a significant role in liberating bound tannins. Those enzymes that showed the highest effect in limiting the adsorption of tannins and in disorganizing the cell wall structure, cellulase and pectinase, did not lead to a desorption of bound tannins, although they still showed a capacity of affecting cell wall structure. The results indicate that enzymes are not able to access those polysaccharides where tannins are bound, thus, they are not a useful tool for desorbing tannins from cell walls. The practical importance implications of these findings are discussed in the manuscript.

## 1. Introduction

The concentration and composition of phenolic compounds are important parameters impacting the quality of wine, since they contribute to key wine organoleptic characteristics, like color and its stability, astringency, bitterness and aroma [1]. Tannins and anthocyanins are the major phenolic compounds extracted from the skins and seeds of the grapes during the red wine vinification process and a high concentration of these compounds in wines has been correlated with higher projected market prices [2].

Many studies have shown that the concentration of phenolic compounds in final wines is much lower than expected, given the concentration in the original grapes [3,4]. In the grapes, phenolic compounds are located inside the cells. Cell-walls act like as a barrier to the extraction of the phenolic compounds present in the grape. Therefore, cell wall degradation will determine the extraction rate/speed of phenolics [5]. There are differences in the extraction rates of anthocyanins and tannins between varieties and this could be due in part to differences in the structure and/or composition of their cell walls [6].

Yet, the concentration of phenolic compounds in wines also depends on another aspect related to grape cell walls. Some studies carried out both in model solutions and in real vinifications have shown that certain phenolic compounds (especially tannins) are susceptible to being bound to the polysaccharides of suspended grape cell walls once they are extracted and this also limits their final concentration in wines. In some studies, carried out on model solutions [7,8], purified cell walls from grape flesh and skin could remove a significant proportion of tannins and the same was verified on the winery scale [9], and it was demonstrated that the presence of suspended plant cell wall material altered the wine composition and the elimination of the suspended grape cell walls during the first steps of the winemaking process increased the net concentration of phenolic compounds, especially tannins, in the final wine.

The different polysaccharide families that form the molecular architecture of the plant cell walls can be targeted by different specific enzymatic activities. Some of these specific hydrolytic enzyme preparations are used in wineries during winemaking, with the aim of extracting components of interest (such as phenolic compounds) from inside cells [5,10]. These enzymes can degrade the cell wall layers and form pores through which internal material (e.g., phenolic compounds) can diffuse. Hydrolytic carbohydrate-active enzymes are normally found naturally within grapes, but their activity is quite low and it is not enough to hydrolyze grape cell walls to a degree effective for winemaking targets [11], so they are added by winemakers. Commercial preparations comprised mainly of pectinases, especially endo-polygalacturonases, pectin-methylesterases and pectin-lyases, and, in addition, cellulases, hemicellulases and proteases, are also commonly present and assist in the disassembly of the components of the grape cell wall during winemaking.

These enzymes may not only help with the transference of phenolic compounds from grape skin cells to the must but also they could block the binding of tannins already in solution to cell walls, probably by degrading their polysaccharide fractions, where tannins bind [8,12]. In previous studies, our research group demonstrated the effect of hydrolytic enzyme addition on the limitation of seed tannin adsorption to cell walls from Monastrell grape skins, observing that pectinases containing pectin-lyase and enzymatic combinations that included this enzyme managed to reduce the adsorption of tannins by the cell walls. These results were in concordance with those of Ruiz-García et al. [13], who demonstrated that the pectic fraction of the cell walls was the one displaying the highest binding capacity for tannins compared to the other cell wall polysaccharide fractions.

However, our interest is also focused in the fate of those tannins already bound to cell wall polysaccharides and the possible role of these enzymes in liberating them into solution. Some previous results indicated that, when trying to liberate the tannins already bound to the cell walls, the ability of the hydrolytic enzymes seemed to be very limited [8,12]. The explanation behind this observation could be a lack of effectivity of the enzymes due to the inability of enzymes to access the polysaccharides of the cell wall, when the tannins are previously bound to them, but this possibility has not been proved.

Therefore, this study has three different objectives: (a) to determine the effect of different hydrolytic enzymes in the interactions between Syrah cell walls and seed tannins; (b) to test if these enzymes could also promote tannin desorption once the cell wall-tannin binding had already taken place in wine-like conditions; and (c) to demonstrate if the enzymes effectively act and modify the cell wall structure in the presence or absence of tannins. Furthermore, identifying which cell wall components are those most affected by the enzymatic action during the adsorption/desorption process can help us to understand the processes taking place during these experiences and this would be very valuable information from a practical point of view.

## 2. Results and Discussion

### 2.1. Tannins Analyzed by HPLC

The first part of our experiment deals with the studies on tannin adsorption to Syrah grape skin cell walls and how the presence of different enzymes may modify this adsorption. Table 1 shows how the concentration of tannins in the solution decreases considerably in the presence of cell walls, confirming the high capacity of cell walls for binding tannins. Approximately 41.8% of the tannins become adsorbed after interaction with cell walls in the absence of enzymes. The extension of cell wall–tannin interactions depends on some factors such as contact time, pH, tannin–cell wall ratio [14], temperature or ethanol concentration [15]. We chose our working conditions (pH 3.5, contact time 90 min and a ratio cell wall/tannin of 6.5) looking for a complete interaction since, according to Renard et al. [14], the adsorption of tannins to cell walls was very fast, reaching a plateau at 20 min, it did not change from pH 2 to 7 and the cell walls could adsorb tannins up to 80% of its initial weight. It is also clear that cell wall composition may also influence the level of adsorption, since in a similar study carried out with Monastrell grape cell walls, an even higher adsorption was observed (54.5%) [16].

The presence of enzymes modified the percentage of adsorbed tannins, clearly suggesting that enzymes are not inhibited by the presence of tannins. The presence of CEL, PEC and XYL enzymes increased the concentration of tannins measured in solution, indicating a lower degree of adsorption as compared to the control experiment without any enzymes. The adsorption values decreased to ca. 28.5% when these enzymes were used. The lowest effect was observed with PME, which only slightly increased the tannin content in solution compared to the control. The lower efficiency of this enzyme in reducing the tannin adsorption by the cell walls may be due to a low capacity to degrade the cell wall polysaccharide network since PME only catalyzes the demethylesterification of galacturonic acid units of pectin. In a similar study using Monastrell cell walls [16], individually added enzymes also caused a decrease in tannin adsorption, pectinase enzymes causing the largest effect, which agrees with the results found for Syrah cell walls.

Regarding the mean degree of polymerization of tannins (mDP), its value decreases in the presence of cell walls from 2.70 to 2.47, reflecting a variation on the polymer/monomer ratio from 9.5 to 7.4. This suggests that the high molecular weight tannins were those that preferentially interact with grape cell walls in the model solution. This has been described and demonstrated in previous studies [8,16]. The addition of the enzymes did not modify this preferential binding. The percentage of tannin galloylation showed a reduction after interaction with cell walls. Tang et al. [17] showed that the number of galloyl groups increases the strength of the binding to macromolecules, so galloylated units were preferably bound to cell walls. The presence of individual enzymes resulted in a slight but significant increase in free galloylated tannins in solution.

The possible effect of enzymes in facilitating the desorption of tannins bound to cell walls was determined after conducting the adsorption test. At this moment, CW-Tan complexes were intensively washed and then reintroduced into a fresh model wine solution and put in contact with the different enzymes. A control without enzyme was also prepared. The percentage of released tannins in the desorption process was calculated by dividing the amount of liberated tannins between the amount of tannins initially retained by the cell walls.

The concentration and composition of the released tannins in the desorption study can be observed in Table 2. Some release of tannins was observed in the control sample (CW-Tan), attributed to the combined action of ethanol concentration in the model solution and stirring. There are other studies that have looked into the effect of different concentrations of ethanol and different temperatures in the release of cell wall-bound proanthocyanidins and anthocyanidins [15,18]. Beaver et al. [15] carried out desorption tests of cell wall-bound tannins and they showed that, by increasing the ethanol from 0% to 15%, the desorption of the tannins previously absorbed by the cell walls increased significantly, therefore, in the conditions in which we carried out this experiment, part of the tannins absorbed by the cell walls can be released just by the action of ethanol. On the other hand, Medina-Plaza et al. [18] performed absorption and desorption tests with cell walls and anthocyanins and they also reported that desorption was highly influenced by the temperature and the concentration of ethanol of the medium, even more than by the composition of the cell wall. They stated that possibly, in a real vinification, the wine punches (when there is already ethanol in the medium) may produce a certain release of the tannins and anthocyanins adsorbed by the cellular material in suspension during the maceration.

The addition of the enzymes CEL and PEC did not entail the release of tannins bound to the CW, since they did not present significant differences with the control. Castro-López et al. [8] performed a similar study carrying out a process of tannin desorption using a commercial enzyme (a mixture of several enzymes similar to those used in this study) and this preparation only managed to release 12% of the tannins bound to the cell walls. The lack of effectiveness of these enzymes in the tests may be due to the fact that they may not able to access the cell wall polysaccharides because of the tannins previously bound to them. Only the PME and XYL treatment liberated significantly higher quantities of tannins than the control. The action of PME in the de-esterification of highly methylated pectins, which have the highest affinity for tannins [19,20,21,22], could explain the higher released soluble tannin content observed when this enzyme was present.

Regarding the composition of the released tannins in the desorption test, they presented a higher mDP and %Gal value than those that remained in solution after the adsorption test, confirming that those tannins bound to cell walls were those more polymerized and galloylated and therefore, their liberation led to an increase in both parameters in the desorption test. The higher increase in mDP and %Gal was mainly observed when the PEC enzyme was used, followed by the use of XYL. The other enzymes did not show significant differences with respect to control treatment. Beaver et al. [15] also observed that the higher molecular weight tannins and those from grape skin were the most susceptible to desorption.

### 2.2. Study of the Effect of the Enzymes on the Cell Wall Structure and its Polysaccharides

#### 2.2.1. Comprehensive Microarray Polymer Profiling (CoMPP) Analysis of Cell Wall Structure

Trying to find a reason for the observed results in the adsorption and desorption tests, one of our first interest was to demonstrate undoubtedly that enzymes, even in the presence of tannins, modify the cell wall structure.

For a better characterization of the cell wall structure and its modification due to the presence of enzymes, the CoMPP methodology, which uses sets of cell wall probes in glycan microarray format, was employed. The CoMPP analysis shows the relative abundance of cell wall polymers extracted with CDTA and NaOH. The treatment of cell walls with CDTA allows the extraction of pectin polymers, whilst NaOH dissolves and extracts mainly hemicelluloses but also unbranched RG-I because it is believed that, in addition to xyloglucan, highly esterified homogalacturonan (HG) and RGI material are coating the hemicellulose-rich fraction [23].

The CoMPP analysis of the cell walls after the adsorption test is shown, as a form of a heatmap, in Table 3. Moreover, the analysis of fresh cell walls (before adding them to the model solution) and the control cell walls, without any enzymes or tannins added, were conducted. The CDTA fraction of the fresh cell wall (CW’) contained mostly pectin polymers and the NaOH fraction extracted hemicellulose polymers and unbranched RGI. Compared with the heatmap observed for Monastrell cell walls [16], less pectin polymers were observed, and the result of this are coincident with the studies of Ortega-Regules et al. [24], who reported a higher content of pectins in Monastrell cell walls than in Syrah cell walls.

The analysis of the CWs that were suspended in the ethanolic model solution showed a higher dissolution of pectin polymers in the CDTA fraction, together with the detection of galactan (mAb LM5) and extensin (mAb LM1) in the NaOH fraction, confirming the effect of ethanol in exposing the cell wall polymers. It is important to point out that the signals from different epitopes were barely influenced by the tannins bound to the cell wall (CW-Tan).

When enzymes were used, a slight decrease in the signals for HG epitopes in the CDTA fraction was observed when CWs were treated with CEL and XYL, whereas a marked decrease in the values for these homogalacturonan (HG) epitopes (mAbs JIM5 and JIM7, LM20 and INRA-RU1) was found when the CWs were treated with PEC. This decrease in HG epitope signal could be attributed to a marked action of this enzyme on the pectin polymers and the diffusion of relatively small oligosaccharides, from within the cell wall, into the model wine-like solution. On another hand, there was a slight increase in the signals of mAb JIM5 for the sample treated with PME, possibly due to its action in the release of free carboxyl groups from esterified HG. A higher presence of de-esterified HG in CW could decrease hydrophobic zones and explain the limitation (although small) of tannin adsorption in the presence of this enzyme, as observed in Table 1.

In the NaOH extract of the cell walls treated with PEC, there was a higher extraction of unbranched RG-I and galactan polymers versus those observed in the untreated CWs. These results are probably due to the unravelling effect of this enzyme on cell wall structure, exposing the hemicellulose layers to the action of ethanol. Similar results were observed by Gao et al. [23,25] studying the cell walls of Cabernet Sauvignon grape pomace isolated after treatment with pectinases enzymes during vinification. Xyloglucan epitope signals slightly decreased when xylanase was present, indicating that, although a xyloglucan depolymerization was promoted by this enzyme, its action was quite limited, probably due to its restricted access to the hemicellulose network, since the pectic matrix had not been degraded by any pectinase [26,27], therefore, this enzyme could only act on those xyloglucans present in that pectic matrix, according to the proposed grape cell wall model by Gao et al. [23] No change from untreated CW was observed in NaOH extract when PME and CEL enzymes were used.

The results of the COMPP analysis confirmed that PEC enzymes, and to a lesser extent CEL, degraded the architecture of the cell walls, whereas XYL and PME barely affected this structure. The two first enzymes produced changes in the wall structure that reduced its capacity to adsorb tannins, explaining the results observed in Table 1.

Trying to determine why enzymes were not effective in desorbing tannins from cell walls, the architecture of the CWs was also studied after the desorption test (Table 4). In the desorption tests, a control cell wall (CW’’) was also included, representing those control cell walls from the adsorption experiment (CW’), subjected for a second time to stirring in a model solution in the absence of enzymes and tannins. This control cell wall (CW”) showed a slight signal decrease for HG and (RG) I (mAb INRA-RU2) and in the xyloglucan signal intensity of the NaOH extract. When the CW-T were studied, only a slight decrease in the mAb INRA-RU2 signal was observed, indicating that the presence of tannins in the cell walls reduces or limits the action of ethanol.

As regards the action of enzymes, and for the CDTA and NaOH extract, the presence of CEL resulted in a slight decrease for HG and xyloglucan signals; however, the presence of PEC resulted in a more important reduction in some HG signals, affecting especially the mAbs JIM7 and LM20 and also in those related to RG I polymers (mAbs INRA-RU1 and INRA-RU2), indicating again, as in the adsorption test, that this enzyme produced a de-pectination that favors a greater exposure of hemicellulose polymers and release of xyloglucan polymers into solution. Only slight changes were observed for the HG signals when PME and XYL were present, although an increase in the signal abundance of xyloglucan polymers was observed for these enzymes and PEC. In the case of PEC, this result could be possibly associated with a higher pectin degradation, making the xyloglucan layer more accessible. Therefore, the analysis of cell wall structure after the desorption test shows that CEL and PEC enzymes also produced a degradation of the cell walls in the desorption experiment, although to a lesser extent than that observed in the absorption tests. The lack of action of the enzymes in liberating the bound tannins seems to indicate that the tannins attached to the cell wall polysaccharides make them very difficult to be reached by enzymes, therefore limiting the degradative action on these polysaccharides and therefore, the liberation of tannins, as indicated by Renard et al. [28]

The small increase in tannins in solution when PME was used could be attributed to a decrease in the strength of tannin adsorption due to pectin demethylation, as stated before. When trying to look for a reason for the slight action of XYL, even when this enzyme barely modified the cell wall structure, we hypothesized that the small size of this enzyme (22 to 29 kDa, according to provider) may allow it to act in some of the tannins bound to hemicelluloses, these interactions being less strong than those with pectins [13]. 

#### 2.2.2. Size Exclusion Chromatography Analysis of the Soluble Polysaccharides Released from Cell Walls

Another way to confirm the effectiveness of the enzymes on the cell wall structure and to try to explain the tannin behavior in the presence of cell walls is the study of the presence of soluble polysaccharides liberated from the cell wall structure in the solution, after the enzyme actuation. Table 5 and Table 6 show the area corresponding to high and medium molecular mass polysaccharides (i.e., those polymers eluting between 12 to 18 min) and the medium to low molecular mass polysaccharides (eluting between 18.01 to 22.5 min) of the liberated polysaccharides after the absorption and desorption tests, conducted in the presence and absence of enzymes. Moreover, to determine if the presence of tannins bound to cell walls could affect the action of enzymes and the liberation of soluble polysaccharides, the action of the enzymes on cell wall degradation was also studied in the absence of tannins.

The results of the adsorption test showed that the model solution itself facilitated the release of polysaccharides from the CW and this is in agreement with the results of the CoMPP analysis that indicated that the ethanolic solution itself caused a change in the cell wall structure and with the results of Beaver et al. [15], who reported the action of ethanol in desorbing tannins bound to cell walls.

In the adsorption test, when XYL and PME were used, the liberation of soluble polysaccharides was only slightly higher than that of the CW without enzymes, which agrees with the previously observed results regarding the lack of effect of the two enzymes in the cell wall structure. The CEL treatment increased the quantities of both high to medium and, especially, low to medium molecular weight polysaccharides, and the use of PEC led to the highest soluble polysaccharide content in solution, this enzyme liberating a significantly high quantity of medium and low molar mass polymers into solution, demonstrating that PEC has a remarkable ability to breakdown and release cell wall polysaccharides into solution, both poly and oligosaccharides. Again, these results coincide with that obtained with CoMPP analyses, where a high de-pectination and de-esterification was observed with this enzyme and explained the largest effect of CEL and PEC enzymes in limiting tannin adsorption due to the decrease of pectin polymers, where tannins preferentially bind. Gao et al. [23,25] also reported the effectiveness of a mix of polygalacturonase and pectin lyase enzymes at removing cell wall pectin-coating layers, providing easier access to the hemicellulose polymers. Furthermore, an increase in the low molecular weight polysaccharide fractions (with a molecular mass range closer to that of the RG II monomer) was found in grape cell walls treated with a polygalacturonase-rich commercial enzyme as performed by Bindon et al. [29] The presence of tannins in the experiment only modified the profile of polysaccharides released, increasing those of a lower molecular weight. The only exception was the experiment where PEC was used, where a decrease in all the different fractions was observed when tannins were present.

In the desorption assay, the hydroalcoholic solution only released a small amount of polysaccharides and no major differences were found with respect to the amounts released for the XYL and PME treatment, related to the effect observed with the CoMPP analysis. CEL treatment led to higher liberation of polysaccharides than XYL and PME, and PEC released the highest quantities of both high and low molecular weight polysaccharides. For all the enzymes, the liberation of the highest molecular mass polysaccharides decreased in the presence of tannins, and this effect was more important when PEC was used. These results agree with those of CoMPP, indicating an action of the enzymes on the cell wall, even when tannins were present. However, and despite these results, the desorption test (Table 2) did not show any liberation of tannins to the medium after the enzyme treatment, except for the PME treatment. It is clear that, compared to the adsorption assay, the liberation of polysaccharides in the desorption test was lower than in the adsorption test. Given that the proportion of tannins to CW material used in this research is far from the maximum capacity of cell wall for tannin adsorption, a capacity that, according to Renard et al. [28], could reach 80% of the initial CW weight, all these results of the desorption experiment point to the hypothesis that enzymes mainly acted on the cell wall polymers free of bound tannins, due to a limitation in their capacity to access effectively to the cell wall polysaccharides where tannins are bound [28].

## 3. Materials and Methods

### 3.1. Grapes

In this study, *Vitis vinifera* L. cv. Syrah grape was sampled from a commercial vineyard located in Jumilla, Murcia (Spain) at technological maturity and were stored at −20 °C until analysis.

### 3.2. Tannins Used in the Adsorption/Desorption Studies

A seed-derived commercial tannin (TanReactive, Agrovin, Alcazar de San Juan, Spain) with a purity of 89.9%, a mean degree of polymerization of 2.7 and a percentage of galloylation of 14.5 was used in the interaction tests. The mean degree of polymerization and the percentage of galloylation were obtained using a phloroglucinolysis method [30]. Purity was assessed spectrophotometrically using the methylcellulose precipitation method [31].

### 3.3. Isolation of the Cell Wall Material as the 70% Ethanol Insoluble Residue

Cell walls (CW) were isolated following the method of de Vries et al. [32] and adapted by Bautista-Ortín et al. [12]

Fresh skins of Syrah grape were used to prepare purified cell walls. Initially skins were separated from the flash frozen grapes using a scalpel. Skins were frozen with liquid nitrogen (−196 °C) and were then ground by hand using a pestle and mortar. The powdered grape skins were suspended in boiling water (100 °C) for 5 min and homogenized at 10,000 rpm for 1 min using a PRO D-Series benchtop homogenizer (PRO Scientific, Oxford, MS, USA). To separate the powered skin from the water solution, the mixture was centrifuged for 10 min at 10,000 rpm. Later on, skin materials were mixed with two parts of ethanol (70% *v*/*v*), agitated (5000 rpm) and heated (50 °C) for 30 min. Next, the mixture was centrifuged (for 10 min at 10.000 rpm) and the supernatant was discarded. Washing with 70% *v*/*v* ethanol was repeated until free sugar was no longer present in the liquid phase (analysis of the soluble sugar in the supernatant using the method of Dubois [33]). Finally, the insoluble solids were washed once with ethanol (96% *v*/*v*), once with acetone (100% *v*/*v*) and were then dried under a stream of air (20 °C) before being stored under darkness.

### 3.4. Adsorption and Desorption Experiments

#### 3.4.1. Adsorption Test

Skin cell walls from grapes were placed in 3 mL tubes and were then mixed with enological tannin previously dissolved in a 2.5 mL wine-like solution (12% ethanol at pH 3.6 adjusted with trifluoroacetic acid). The final concentration in the mixture was 13 mg/mL for the cell walls and was 2 mg/mL for the seed tannin. Next, hydrolytic enzymes were added to all tubes, except for the control (CW + Tan) treatment.

The following pure enzymes supplied from Sigma-Aldrich (St. Louis, MO, USA) were used individually; (1) cellulase (EC 3.2.1.4, CEL, 100 mg/L) from *Aspergillus niger*, (2) pectin-methylesterase (EC 3.1.1.11, PME, 25 mg/L), (3) a pectinase that comprise two activities: pectin-lyase plus polygalacturonase (EC 4.2.2.10 and EC 3.2.1.15, PEC, 100 mg/L) from *Aspergillus japonicus* and (4) a xylanase (XYL) (EC 3.2.1.8, XYL, 50 mg/L) from *Thermomyces lanuginosus*.

Next, tubes were shaken first in a vortex and then in an orbital shaker (300 rpm) for 90 min at ambient temperature. During this period, the binding between the tannin and the suspended grape cell walls are believed to occurred in the presence and absence of enzymes. Therefore, it would be possible to verify if the presence of enzymes interfered with the interaction between the tannins and the grape cell walls.

Six replicates were carried out for each treatment, three of them were redissolved in methanol and used for the determination of tannins by phloroglucinolysis. The other three were redissolved in MiliQ water for the analysis of the molar mass distribution of the released polysaccharides by SEC. The tannin–cell wall complexes were precipitated (70% ethanol, followed by absolute ethanol and acetone) to obtain AIR powders.

#### 3.4.2. Desorption Test

For the desorption test, an adsorption process between tannins and cell walls was initially performed, similar to that described previously (in 3.4.1.) After allowing for the interaction to take place, the supernatant (which contained the tannins that had not interacted with the cell walls) was discarded and the cell wall–proanthocyanidin complexes were redissolved in 2.5 mL of the model wine solution. Different model wine solutions were used which also contained different pure enzymes, as performed for the adsorption test. Desorption tests in the absence of enzymes (CW-Tan) and in the absence of tannin and enzymes (CW’’) were also carried out.

The tubes were shaken at 300 rpm for 90 min in an orbital shaker. The supernatants were analyzed, under the same conditions as the adsorption tests, to monitor desorption of tannins from cell walls occurring in the different treatments.

Six replicates were carried out for each treatment, three of them were dissolved in methanol and used for determination of tannins by phloroglucinolysis. The other three were dissolved in ultrapure water for the determination of the molar mass distribution of the released polysaccharides by SEC. Tannin–cell wall complexes were prepared by precipitation in 70% ethanol, followed by absolute ethanol and acetone, in order to obtain AIR powders.

### 3.5. Analysis of Tannins Using the Phloroglucinolysis Reagent

The phloroglucinolysis reagent was used to determinate the tannin concentration and composition of the methanolic extract obtained from the adsorption/desorption processes. The method was described by Kennedy and Jones [30] and it was followed with some modifications.

Initially, 50 µL of the methanolic extract of the tannins were mixed with 50 µL of the phloroglucinolysis reagent. This was prepared by dissolving phloroglucinol (Sigma, Aldrich, St. Louis, MO, USA) (100 g/L) and ascorbic acid (Sigma, Aldrich, St. Louis, MO, USA) (20 g/L) in a 2 N hydrochloric acid solution in methanol. The mixture was heated in a water bath at 50 °C for 20 min. To quench the reaction, 100 µL of an aqueous sodium acetate solution (0.2 M) was then added.

Tannins were estimated using their response factors relative to (+)-catechin, used as the quantitative standard. Using the phloroglucinolysis reagent, the total tannin content, the apparent mDP and the percentage of each constitutive unit were then determined. The sum of the flavan-3-ol monomer subunits and the phloroglucinol adducts (mol) divided by the sum of all flavan-3-ol monomers (mol) is the mDP. The standards (+)-catechin, (-)-epicatechin, (-)-epicatechin gallate and (-)-epigallocatechin were obtained from Extrasyntese (Genay, France).

HPLC analysis conditions were previously described [4]. Briefly, 10 µl of each sample were analyzed using an Atlantis dC18 column (250 × 4.6 mm, 5 µm packing) and a pre-column of the same material (20 mm × 4.6 mm, 5 µm packing). The flow rate was 0.8 mL/min and the temperature of the oven was set at 30 °C. The mobile phase was composed of two solvents: a solution of formic acid in water (2% *v*/*v*) (solvent A) and a solution of the solvent A in acetonitrile (20:80 *v*/*v*) (solvent B). Elution began with 100% A and 0% B for 5 min. Then a linear gradient from 0 to 10% B in 30 min and gradient from 10 to 20% in 30 min, followed by washing and re-equilibration of the column.

### 3.6. Analysis of Soluble Polysaccharides by Size Exclusion Chromatography

The molecular mass distribution of soluble polysaccharides liberated from cell walls in hydroalcoholic solutions in the presence and absence of enzymes was analyzed by size exclusion chromatography (SEC). The concentrated samples were re-dissolved in 250 µL of MiliQ water, and a 20 µL volume of this solution was injected into a flow stream of 1 mL/min of LiNO3 (0.1 M) that runs through two columns Shodex Ohpak KB-803 and KB-805 (0.8 cm × 30 cm, Showa Denko K. K., Japan) connected in series. A refractometer was used as the detector (Waters 2414) system. The molar mass was determined using a calibration curve made with a special molar mass polymer kit (P-400, PM = 380.000; P- 200, PM = 186.000; P-100, PM = 100.000; P-50, PM = 48.000; P-20, PM = 23.700; P-10, PM = 12.200; P-5, PM = 5.800; Showa Denko K.K., Japan).

### 3.7. Cell Wall Profiling Using Glycan Microarrays

For the determination of cell wall composition by virtue of epitope composition using various plant cell wall antibodies and probes, 10 mg of each cell wall sample was analyzed using the CoMPP (Comprehensive Microarray Polymer Profiling) glycan microarray technique described in Osete-Alcaraz et al. [16] This technique involves first performing CDTA (diamino-cyclo-hexane-tetra-acetic acid) cell wall extraction, which produces a pectin-rich fraction followed by 4N NaOH, producing a hemicellulose-rich fraction. The two fraction classes were printed on nitrocellulose membranes and probed individually with 26 different monoclonal antibodies (mAbs) and carbohydrate binding modules (CBMs) as described in Moller et al. [34] A mean spot signal was calculated and normalized to the highest signal in the dataset, being set to 100, applying a cut-off of 5.

### 3.8. Statistical Analysis

The statistical analysis of the results was made using the statistical package Minitab 18. Initially an analysis of variance (ANOVA) was made to determine differences among samples. When there were significant differences, Tukey’s test was used to separate the means with a confidence level of 95% (P-value of 0.05).

## 4. Conclusions

Syrah cell walls present a high capacity of binding tannins (ca. 41%), although they are lower than that reported for Monastrell cell walls, indicating that the cell wall–tannin affinity is influenced by the grape variety. In real winemaking, this should be taken into account, since this affinity will affect the extension of the tannins that will keep in solution during the process.

All the enzymes used, except PME, reduced the absorption of tannins by the cell walls. We have clearly demonstrated that this reduction is due to the effect of enzymes on the cell wall architecture, since there is a degradation of the polysaccharide network where tannins bind and there is a liberation of soluble polysaccharides to the medium. Although the enzymes used differently affected the characteristics and extension of the cell wall breakdown, it did not result in clear differences in their ability to limit adsorption. PME was the enzyme that produced the least degradation in the cell walls, only producing the demethylation of the HG of the pectins, being insufficient to reduce the tannin adsorption capacity of the cell walls.

On the other hand, CEL and PEC, despite extensively degrading CW in the desorption tests (although to a lesser extent than in adsorption tests), did not show any tannin desorption capacity from cell walls, indicating that the enzymes were not able to access and degrade the polysaccharides where tannins are strongly bound.

From a practical point of view, the enzymes tested in this study could be used in vinifications since they will favor grape cell wall degradation and the extraction of the phenolics located inside the cells and, at the same time, they will limit the adsorption of tannins to the suspended cell walls. Moreover, they will increase (especially CEL and PEC) the content of soluble polysaccharides in wines, positively affecting wine sensory properties. However, those tannins bound to cell walls cannot be enzymatically liberated and will be lost, together with the suspended vegetal material, during clarification processes and rackings. Some other options need to be sought for limiting these losses of wine tannins, especially looking for processes able to limit the presence of suspended cell wall material during winemaking.

## Figures and Tables

**Table 1:** Concentration and composition of tannins that remained in solution in the adsorption test.

Samples	TT (mg/L)	% Adsorption	mDP	%Gal
**Tannin**	1788.4 c *		2.70 b	14.5 d
**CW + Tan**	1040.3 a	41.8	2.49 a	12.3 a
**CW + Tan + CEL**	1274.5 b	28.7	2.48 a	12.8 bc
**CW + Tan + PME**	1179.2 ab	34.1	2.49 a	12.6 b
**CW + Tan + PEC**	1272.1 b	28.9	2.47 a	12.9 c
**CW + Tan + XYL**	1278.4 b	28.5	2.47 a	12.8 bc

CW concentration in the solution: 13 mg/mL. Abbreviations: Tan: Tannin; CW: Cell-wall; CEL: Cellulase; PME: Pectin-methylesterase; PEC: Pectinase; XYL: Xylanase; TT: Total Tannin; mDP: Mean degree of polymerization; % Gal: % Galloylation. * Different letters in the same column mean statistically significant differences (*p* < 0.05).

**Table 2:** Concentration and composition of released tannins into solution measured by phloroglucinolysis in the desorption test.

Samples	TT (mg/L)	% Released	mDP	%Gal
**CW-Tan**	186.0 a *	24.9	3.05 ab	15.3 ab
**CW-Tan + CEL**	185.6 a	24.8	3.00 ab	14.9 a
**CW-Tan + PME**	227.4 b	30.4	2.99 a	14.9 a
**CW-Tan + PEC**	166.7 a	22.3	3.24 c	16.3 c
**CW-Tan + XYL**	219.7 b	29.4	3.09 b	15.4 b

Abbreviations: Tan: Tannin; CW: Cell-wall; CEL: Cellulase; PME: Pectin-methylesterase; PEC: Pectinase; XYL: Xylanase; TT: Total Tannin; mDP: Mean degree of polymerization; %Gal: percentage of galloylation. * Different letters in the same column mean statistically significant differences (*p* < 0.05).

**Table 3:** Heatmap of epitope abundance (0–100) found in the pectin (CDTA) and hemicellulose (NaOH) fractions extracted from the grape skin cell walls of fresh (CW’) and treated (CW) grapes, after interaction with a tannin in the absence or presence of enzymes added individually to grape cell walls in an adsorption test.

Fractions	Samples	HG partially/de-esterifies (mAb JIM5)	HG partially esterifies (mAb JIM7)	HG partially/de-esterifies (mAb LM8)	HG partially/de-esterifies (mAb LM19)	HG partially esterifies (mAb LM20)	HG Ca2+ crosslinked (mAb 2F4)	Xylogalacturonan (mAb LM8)	Backbone of RG I (mAb INRA-RU1)	Backbone of RG I (mAb INRA-RU2)	(1→4)-β-D-galactan (mAb LM5)	Feruloylated (1→4)-β-D-galactan (mAb LM9)	Linearised (1→5)-α-L-arabinan (mAb LM13)	(1→4)-β-D-mannan (mAb LM21)	(1→4)-β-D-mannan/galactomannan (mAb LM22)	(1→3)-β-D-glucan (mAb BS-400-2)	Xyloglucan (XXXG motif) (mAb LM15)	Xyloglucan (mAb LM25)	(1→4)-β-D-xylan (mAb LM10)	(1→4)-β-D-xylan/arabinoxylan (mAb LM11)	Cellulose (Cristalline < 9 (mAb CBM3a)	Extensin (mAb LM1)	Extensin (mAb JIM11)	Extensin (mAb JIM20)	AGP (mAb JIM8)	AGP (mAb JIM13)	AGP (mAb JIM14)	AGP, β-linked GlcA (mAb LM2)
**CDTA**	**CW’**	31	49	9	0	47	0	0	10	13	0	0	0	0	0	0	0	0	0	0	0	0	0	0	0	0	0	0
	**CW **	43	59	15	8	51	0	0	17	26	0	0	0	0	0	0	0	0	0	0	0	0	0	0	0	0	0	0
	**CW + Tan**	46	65	18	10	64	0	0	19	26	0	0	0	0	0	0	0	0	0	0	0	0	0	0	0	0	0	0
	**CW + Tan + CEL**	36	47	14	7	42	0	0	16	26	0	0	0	0	0	0	0	0	0	0	0	0	0	0	0	0	0	0
	**CW + Tan + PME **	58	73	21	9	66	0	0	20	29	0	0	0	0	0	0	0	0	0	0	0	0	0	0	0	0	0	0
	**CW + Tan + PEC**	37	26	20	7	13	0	0	12	25	0	0	0	0	0	0	0	0	0	0	0	0	0	0	0	0	0	0
	**CW + Tan + XYL**	39	63	15	8	56	0	0	16	22	0	0	0	0	0	0	0	0	0	0	0	0	0	0	0	0	0	0
**NaOH**	**CW’**	0	0	0	0	0	0	0	11	7	0	0	0	0	0	0	90	87	0	0	0	0	0	0	0	0	0	0
	**CW **	0	0	0	0	0	0	0	12	9	6	0	0	2	0	0	81	81	0	0	0	0	11	0	0	0	0	0
	**CW + Tan**	0	0	0	0	0	0	0	10	0	4	0	0	0	0	0	84	84	0	0	0	0	11	0	0	0	0	0
	**CW + Tan + CEL**	0	0	0	0	0	0	0	10	8	2	0	0	0	0	0	80	80	0	0	0	0	10	0	0	0	0	0
	**CW + Tan + PME **	0	0	0	0	0	0	0	12	7	0	0	0	0	0	0	83	81	0	0	0	0	12	0	0	0	0	0
	**CW + Tan + PEC**	0	0	0	0	0	0	0	14	22	8	0	0	0	0	0	79	77	0	0	0	0	12	0	0	0	0	0
	**CW + Tan + XYL**	0	0	0	0	0	0	0	11	2	0	0	0	0	0	0	76	74	0	0	0	0	11	0	0	0	0	0

**Table 4:** Heatmap of epitope abundance (0–100) found in the pectin (CDTA) and hemicellulose (NaOH) fractions extracted from the grape skin cell walls treated (CW’’) after interaction with a tannin in the absence or presence of enzymes added individually to grape cell walls in a desorption test.

Fractions	Samples	HG partially/de-esterifies (mAb JIM5)	HG partially esterifies (mAb JIM7)	HG partially/de-esterifies (mAb LM8)	HG partially/de-esterifies (mAb LM19)	HG partially esterifies (mAb LM20)	HG Ca2+ crosslinked (mAb 2F4)	Xylogalacturonan (mAb LM8)	Backbone of RG I (mAb INRA-RU1)	Backbone of RG I (mAb INRA-RU2)	(1→4)-β-D-galactan (mAb LM5)	Feruloylated (1→4)-β-D-galactan (mAb LM9)	Linearised (1→5)-α-L-arabinan (mAb LM13)	(1→4)-β-D-mannan (mAb LM21)	(1→4)-β-D-mannan/galactomannan (mAb LM22)	(1→3)-β-D-glucan (mAb BS-400-2)	Xyloglucan (XXXG motif) (mAb LM15)	Xyloglucan (mAb LM25)	(1→4)-β-D-xylan (mAb LM10)	(1→4)-β-D-xylan/arabinoxylan (mAb LM11)	Cellulose (Cristalline < 9 (mAb CBM3a)	Extensin (mAb LM1)	Extensin (mAb JIM11)	Extensin (mAb JIM20)	AGP (mAb JIM8)	AGP (mAb JIM13)	AGP (mAb JIM14)	AGP, β-linked GlcA (mAb LM2)
**CDTA**	**CW’’**	31	54	11	0	46	0	0	12	23	0	0	0	0	0	0	0	0	0	0	0	0	0	0	0	0	0	0
	**CW-Tan **	36	56	11	0	49	0	0	11	16	0	0	0	0	0	0	0	0	0	0	0	0	0	0	0	0	0	0
	**CW-Tan + CEL**	27	46	8	0	37	0	0	10	18	0	0	0	0	0	0	0	0	0	0	0	0	0	0	0	0	0	0
	**CW-Tan + PME **	40	58	13	0	53	0	0	14	22	0	0	0	0	0	0	0	0	0	0	0	0	0	0	0	0	0	0
	**CW-Tan + PEC **	37	20	13	0	4	0	0	7	11	0	0	0	0	0	0	0	0	0	0	0	0	0	0	0	0	0	0
	**CW-Tan + XYL **	40	51	11	0	47	0	0	15	23	0	0	0	0	0	0	0	0	0	0	0	0	0	0	0	0	0	0
**NaOH**	**CW’’**	0	0	0	0	0	0	0	12	6	0	0	0	0	0	0	76	71	0	0	0	0	6	0	0	0	0	0
	**CW-Tan **	0	0	0	0	0	0	0	11	4	0	0	0	0	0	0	77	76	0	0	0	0	6	0	0	0	0	0
	**CW-Tan + CEL **	0	0	0	0	0	0	0	14	8	0	0	0	0	0	0	70	69	0	0	0	0	0	0	0	0	0	0
	**CW-Tan + PME **	0	0	0	0	0	0	0	14	6	0	0	0	0	0	0	87	84	0	0	0	0	4	0	0	0	0	0
	**CW-Tan + PEC**	0	0	0	0	0	0	0	14	16	0	0	0	0	0	0	87	82	0	0	0	0	6	0	0	0	0	0
	**CW-Tan + XYL**	0	0	0	0	0	0	0	13	6	0	0	0	0	0	0	85	82	0	0	0	0	7	0	0	0	0	0

**Table 5:** Total area, high and medium molecular mass polysaccharides (those eluting from 12 to 18 min) and low to medium molecular mass polysaccharides (eluting from 18.1 to 22.5 min) measured in the size exclusion chromatography analysis for the polysaccharides liberated from the cell-walls in the adsorption tests.

Samples	Area (12–22.5 min)	Area (12–18 min)	Area (18.01–22.5 min)
**CW**	75.04	53.91	21.03
**Tan**	18.51	−5.12	23.64
**CW + Tan**	79.30	39.53	39.68
**CW + CEL**	168.93	75.08	93.53
**CW + Tan + CEL**	164.65	56.92	107.61
**CW + PME**	91.50	63.37	27.93
**CW + Tan + PME**	87.41	41.44	45.87
**CW + PEC**	675.30	59.24	614.49
**CW + Tan + PEC**	461.69	17.65	443.09
**CW + XYL**	91.87	69.66	22.03
**CW + Tan + XYL**	92.71	44.50	48.11

Abbreviations: Tan: Tannin; CW: Cell-wall; CEL Cellulase; PME: Pectin-Methylesterase; PEC: Petinase; XYL: Xylanase.

**Table 6:** Total area, height and medium molecular mass polysaccharides (those eluting from 12 to 18 min) and low to medium molecular mass polysaccharides (eluting from 18.1 to 22.5 min) measured in the size exclusion chromatography analysis for the polysaccharides liberated from the cell walls in the desorption tests.

Samples	Area (12–22.5 min)	Area (12–18 min)	Area (18.01–22.5 min)
**CW-Tan**	13.91	11.78	2.11
**CW’’ + CEL**	99.07	29.24	69.76
**CW-Tan + CEL**	67.39	13.00	54.36
**CW’’ + PME**	19.15	13.63	5.50
**CW-Tan + PME**	13.23	5.04	8.19
**CW’’ + PEC**	450.60	31.10	418.39
**CW-Tan + PEC**	337.09	6.06	330.45
**CW’’ + XYL**	17.15	14.20	2.93
**CW-Tan + XYL**	16.44	8.77	7.66

Abbreviations: Tan: Tannin; CW: Cell-wall; CW’’: control cell walls from the adsorption experiment (CW’) subjected for a second time to stirring in a model solution.; CEL Cellulase; PME: Pectin-Methylesterase; PEC: Pectinase; XYL: Xylanase.
